# Boronic acids for sensing and other applications - a mini-review of papers published in 2013

**DOI:** 10.1186/s13065-014-0060-5

**Published:** 2014-10-18

**Authors:** Karel Lacina, Petr Skládal, Tony D James

**Affiliations:** CEITEC, Masaryk University, Kamenice 5, 62500 Brno, Czech Republic; Department of Chemistry, University of Bath, Claverton Down, Bath, BA2 7AY UK; Department of Biochemistry, Faculty of Science, Masaryk University, Kamenice 5, 62500 Brno, Czech Republic

**Keywords:** Boronic acid, Sensing, Detection of glucose, Diol, Catechol, Fluoride, Reactive oxygen species

## Abstract

Boronic acids are increasingly utilised in diverse areas of research. Including the interactions of boronic acids with diols and strong Lewis bases as fluoride or cyanide anions, which leads to their utility in various sensing applications. The sensing applications can be homogeneous assays or heterogeneous detection. Detection can be at the interface of the sensing material or within the bulk sample. Furthermore, the key interaction of boronic acids with diols allows utilisation in various areas ranging from biological labelling, protein manipulation and modification, separation and the development of therapeutics. All the above uses and applications are covered by this mini-review of papers published during 2013.

## Introduction

**Figure 1:**
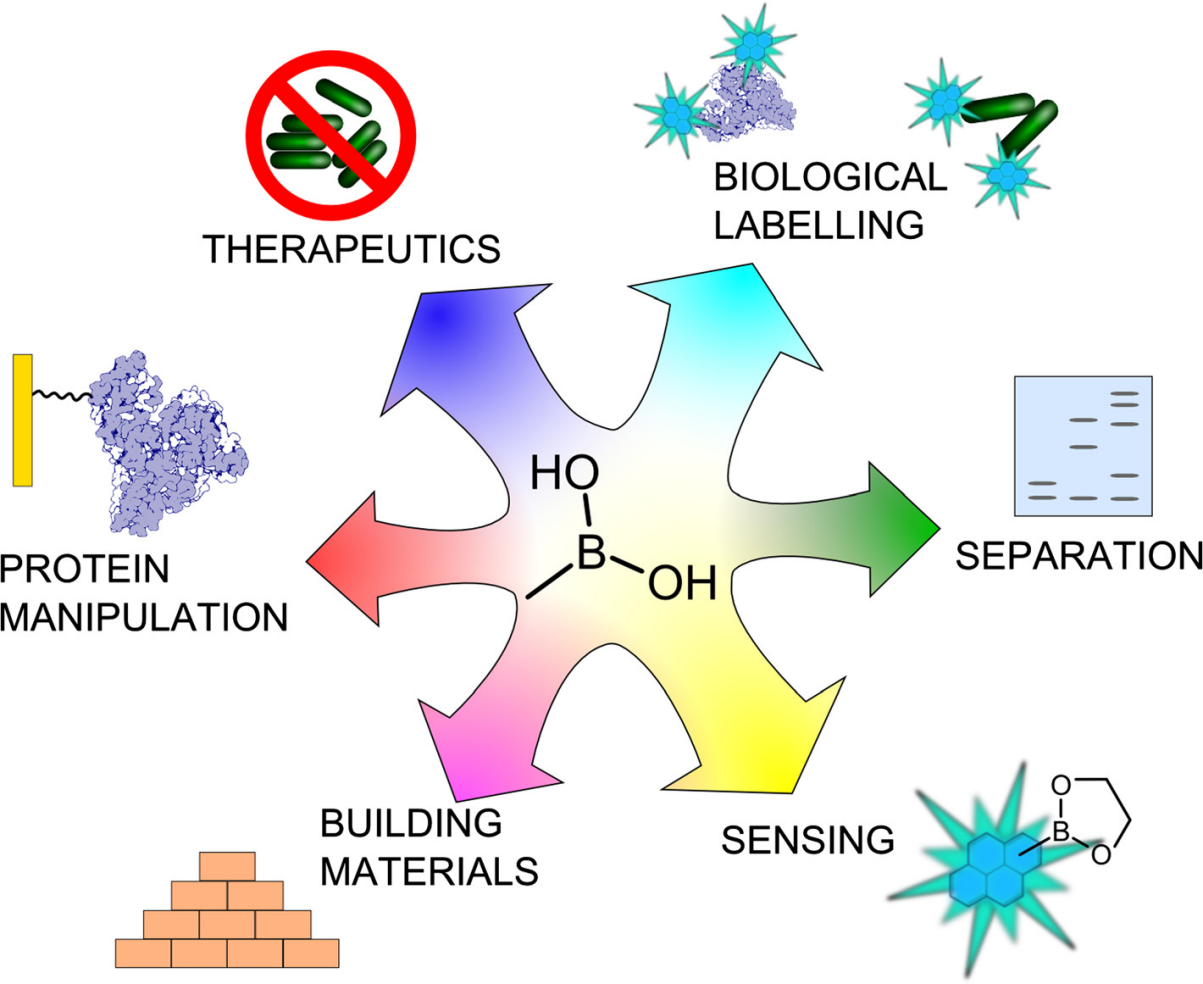
**Diverse usage and applications of boronic acids.**

The following article is a mini review on boronic acids for sensing and other applications for papers published in 2013. It is important to note that the review does not cover any synthetic applications of boronic acids for publications on these areas you are directed to some excellent recent reviews [1–3]. This text does however cover the diverse range of uses and applications for boronic acids from therapeutics to separation technologies (Figure 1).

During 2013 the established area of synthetic receptors for low molecular compounds was further developed, in particular some novel detection methodologies were introduced. However, an area of particular growth was the interaction of boronic acids with proteins, their manipulation and cell labelling. Boronic acid were also used for electrophoresis of glycated molecules. They were also employed as building materials for microparticles for analytical methods and in polymers for the controlled release of insulin.

## Reviews

Boronic acid molecules as the building block for a diverse range of sensing systems has been the central theme for several review papers during the course of the 2013. A general review on all aspects of boronic acid interactions with *cis*-diols and sensing applications [4] and a summary of sensing with multivalent boronic acid sensor molecules discussing methods to improve selectivity towards specific analytes [5] were published. Other reviews dealt with the use of boronic acids as biochemical tools for various purposes, including the interference in signalling pathways, enzyme inhibition and cell delivery systems [6]. While an overview of organoborons [7] and boronic acid-functionalised materials [8], demonstrates their crucial role in carbohydrate chemistry and glycobiology, especially the areas of analysis, separation, protection, and activation.

Boronic acid-containing hydrogels are important intelligent materials. The introduction of boronic acid functionality to these hydrogels allows them to exhibit many interesting properties, such as glucose-selectivity/sensitivity, reversible binding and self-healing ability. The application with an emphasis on biomedical areas – i.e. the design of various glucose sensors and self-regulated insulin delivery devices have been described in detail [9].

However, pure sensing applications represent the major impetus of boronic acid research. Reviews summarising fluorescence [10,11] and electrochemically active derivatives of boronic acid [12] have been compiled. Boronic acid sensors with practical applications were part of a review on subcutaneous *in vivo* glucose monitoring [13,14].

### Sensing in homogeneous phase with synthetic receptors

**Figure 2:**
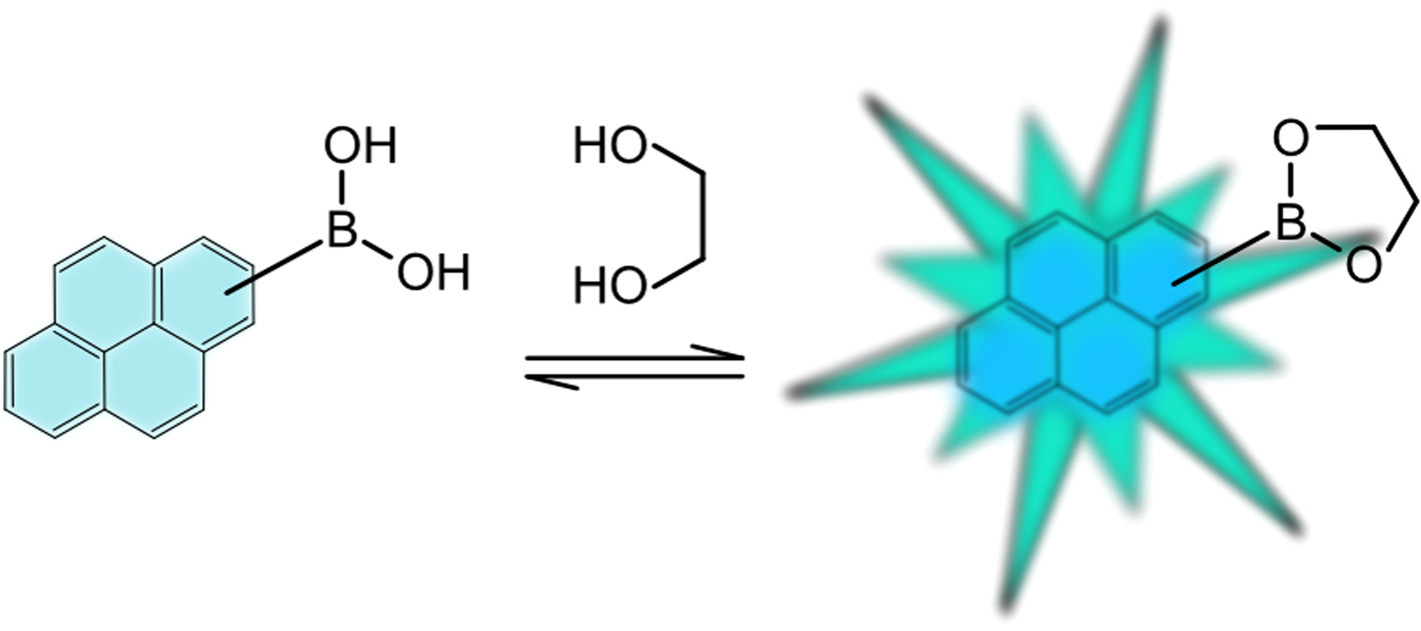
**Boronic acid-based molecular sensors for homogeneous optical detection of diols.**

The most common sensing format is the homogeneous assay (Figure 2). Two interacting partners – analyte and the molecular sensor are present in solution. The sensor molecules containing a boronic acid and reporting unit are prepared to match the sensing application and analytical environment. Interactions between the analyte and the sensor result in the change in the physico-chemical properties of the reporter.

#### Carbohydrates

The most pursued molecular sensor based on boronic acid is a sensor for monosaccharides and in particular glucose. Several reports dealing with the synthesis and characterisation of novel fluorescent recognition systems with new mechanisms are being actively pursued. For example pyrene-based boronic acids were shown to bind glucose [15] and monosaccharides [16].

The well-known and elegant Alizarin Red S (ARS) - phenylboronic acid displacement assay developed by Binghe Wang [17] was adapted using a fluorescent dye and a quencher - boronic acid appended viologen. The quenched fluorescence of the dye was recovered upon boronic acid-glucose interaction [18].

**Figure 3:**
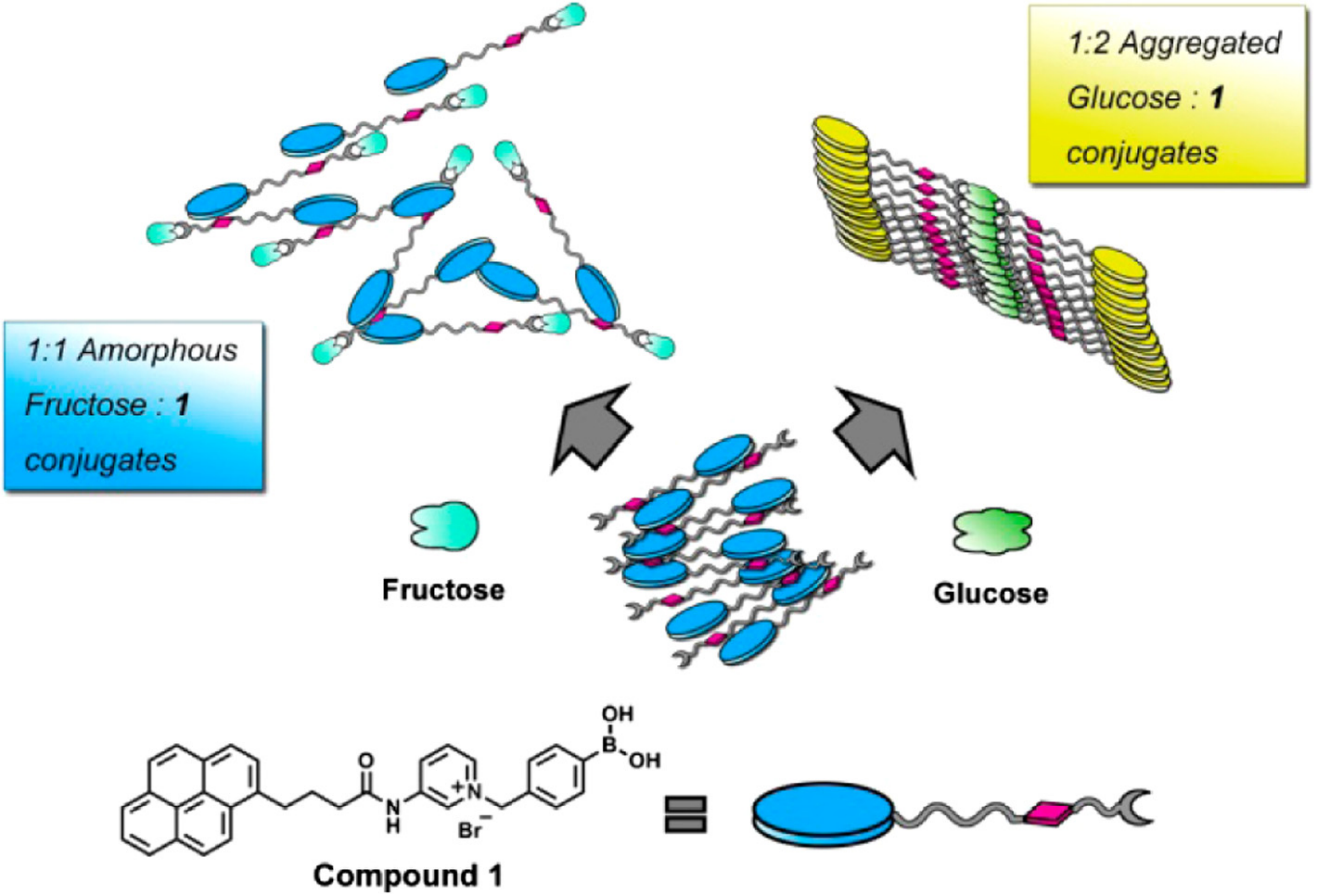
**Molecular sensor forms amorphous conjugates in the presence of D-fructose.** Highly structured and fluorescent conjugates are formed in the presence of D-glucose. (Adapted from ref. [19]).

The problem associated with the higher affinity of boronic acids towards D-fructose over D-glucose was addressed in the elegant work by James and Jiang (Figure 3). Higher specificity of boronic acid towards glucose was achieved using the stoichiometry of “glucose” and “fructose” binding. The pyrene-boronic acid derivative produced amorphous 1:1 conjugates in the presence of D-fructose. While highly ordered and fluorescent 2:1 conjugates were obtained in the presence of D-glucose. With this system both boronic acid-diol interactions and the π-stacking and aggregation of pyrene units were involved in the selectivity of the sensing mechanism. Moreover D-fructose in a mixture of D-fructose and D-glucose could be “knocked-out” by adding phenylboronic acid since it interacts strongly (selectively binds) with D-fructose [19].

**Figure 4:**
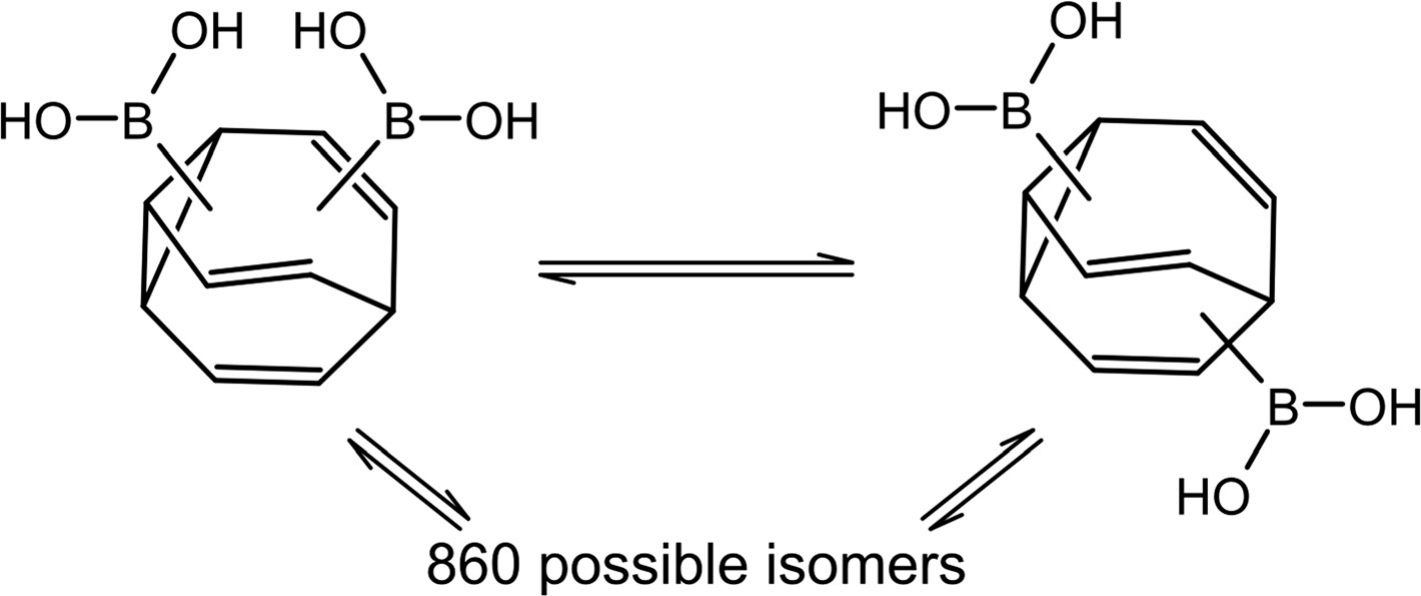
**Boronic acid appended bullvalene can form a multitude of isomers allowing construction of a self-contained sensor array** [20]**.**

An interesting molecule with great potential, is boronic acid modified bullvalene and its ability to isomerise into different shapes was utilised for polyol detection (Figure 4). The labile structure of the sensor molecule could be frozen by addition of polyol and the changes followed by ^13^C NMR. The NMR spectrum was used to develop a “finger-print” barcode. The sensor molecule could be considered as a self-contained sensor array. The interaction with ten diverse polyols in DMSO/phosphate buffer was investigated. Mixtures of polyols could be analysed with this sensor molecule and the strongest binding analyte dominated the corresponding read-out. It was proposed that NMR can be used as a noninvasive technique for *in vivo* sensing [20].

Many fluorogenic units are used for reporting the interactions of diols with boronic acids. The fluorophores used include pyrene [15,16,19], coumarin [21], naphthalene [22] and BODIPY [23]. Furthermore multi-colorimetric sensor arrays were developed using boronic acid-containing thin films combined with three anionic dyes for saccharide detection. The binding of saccharides was monitored *via* changes of colour [24].

A boronic acid-based sensor system was used for the detection and labelling of bacteria [22]. A change in pH caused by the boronic acid-fructose interaction was followed during the fermentation process. The pH dependent change of colour by the indicator rhodamine B resulted in a measurable signal “out-put” [25].

**Figure 5:**
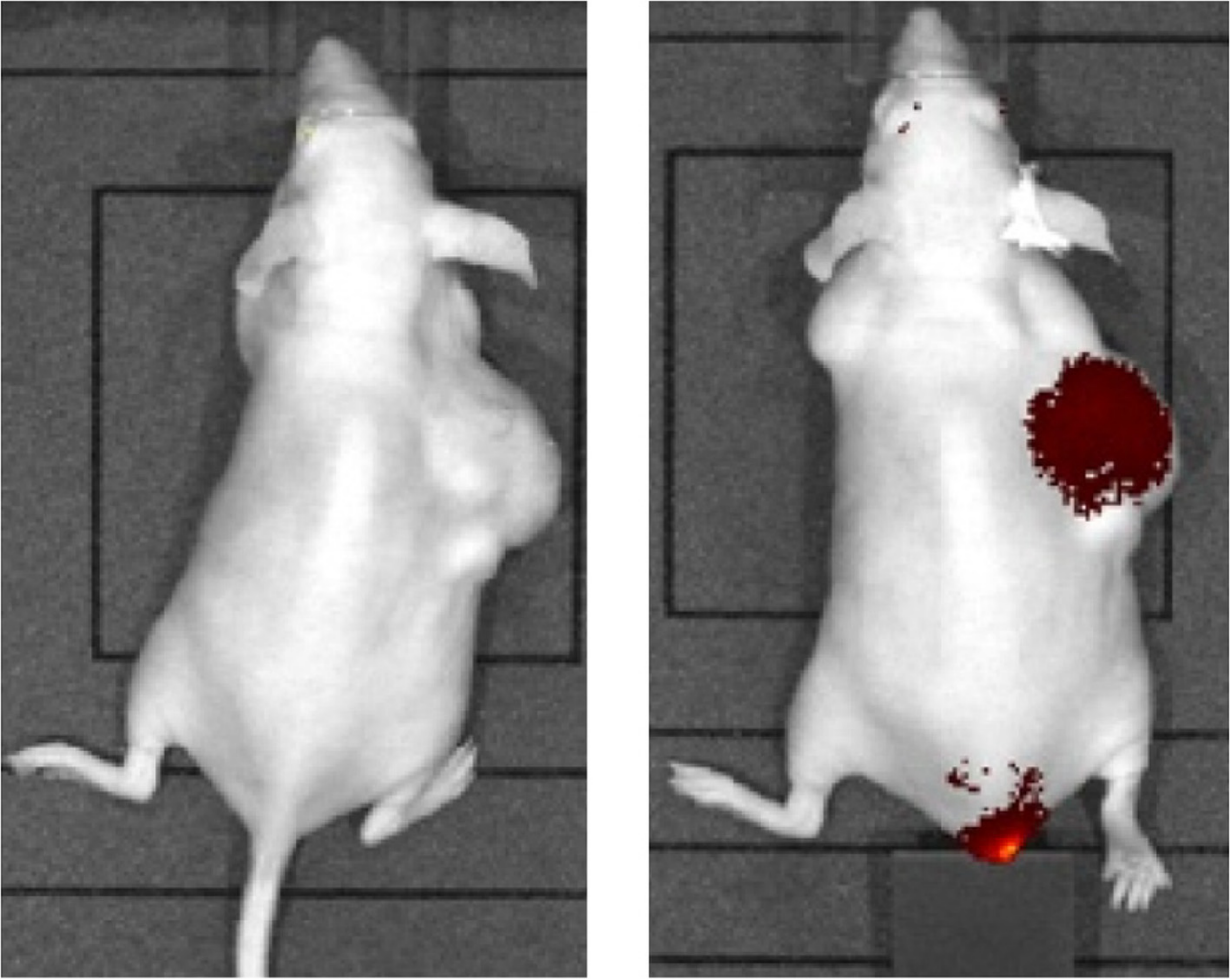
**Optical imaging of xenograft tumour by specific boronolectin-fluorophore, left: mouse before imaging agent injection; right: mouse 24 h after tail vein injection of the contrast agent showing almost exclusive delivery to the tumour site.** (Adapted from ref. [27]).

Boronic acid – carbohydrate interactions were also employed for the fluorescence visualisation of tumours. Sensor system prepared using a peptide connected with two boronic acid groups (anthracene based sensors [4]) exhibited high specificity towards sialyl Lewis X and was used for the selective labelling of cell-surface glycans of human hepatic cancer cells [26]. Over-expressed carbohydrate-based carcinoma biomarker, sialyl Lewis X was selectively labelled in the mouse xenograft tumour via this novel boronolectin-fluorophore (Figure 5) [27].

#### Catechol derivatives

**Figure 6:**
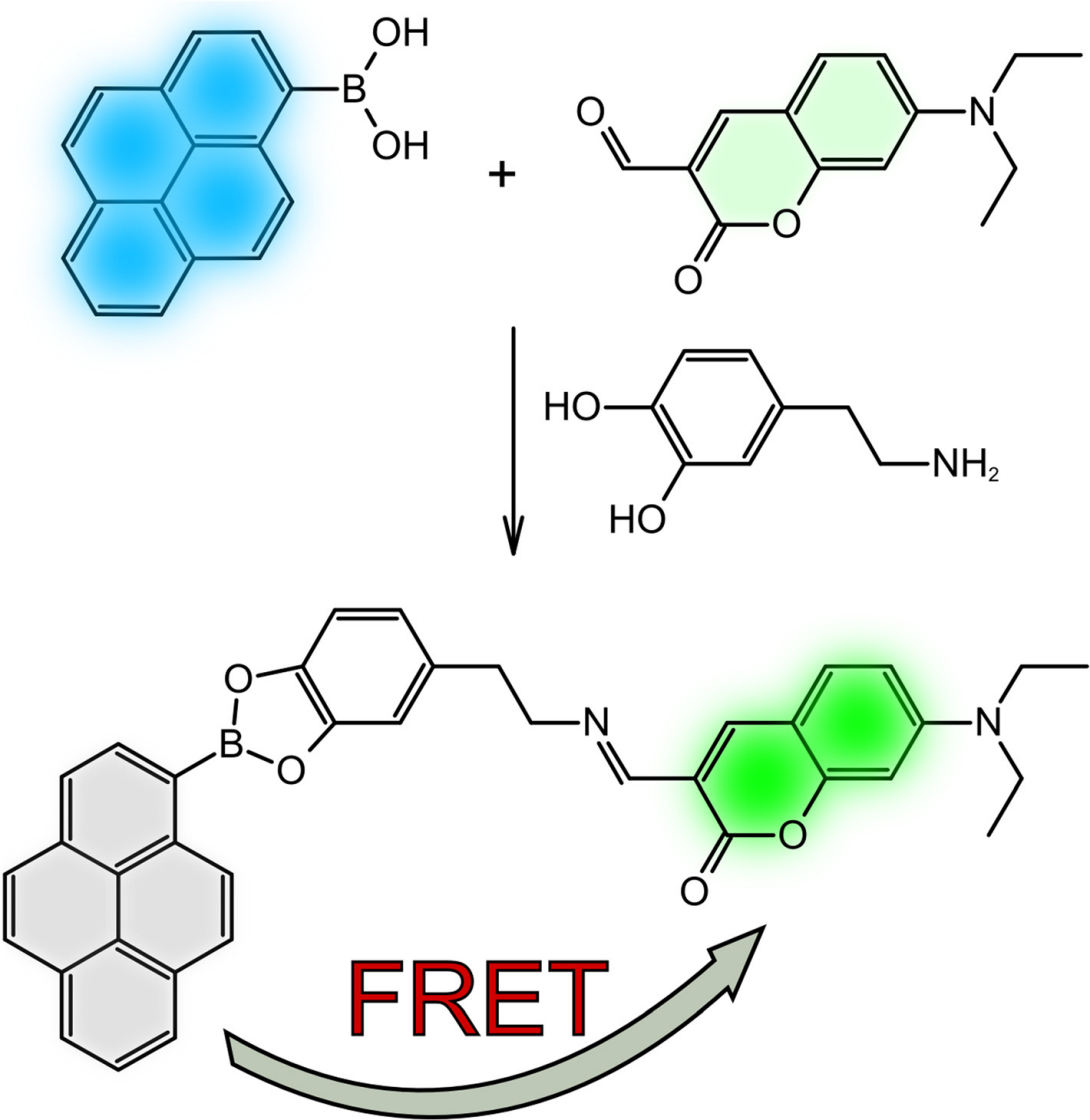
**Schematic visualisation of the detection system for catecholamine derivatives combining boronic acid-diol, aldehyde-amine interactions and the FRET process** [28]**.**

Aromatic *cis*-diols/catechol derivatives are another group of compounds that strongly bind to boronic acid receptors. A detection system based on the FRET between two separate fluorophore containing receptor units permitted the selective detection of catecholamine derivatives (Figure 6). In the presence of the catechol analyte the two sensor molecules – boronic acid and aldehyde are brought into close proximity and FRET was observed [28].

**Figure 7:**
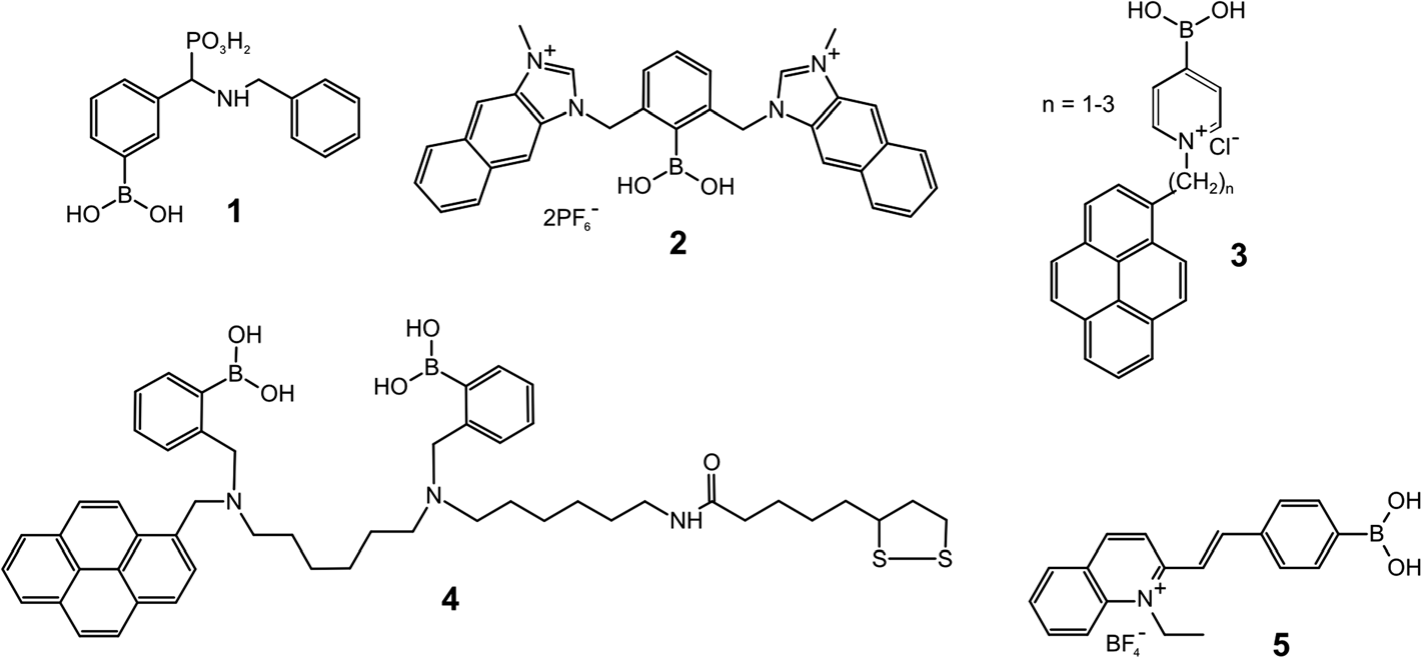
**Structures of selected boronic acid derivatives.**

Another fluorescent sensor combining boronic acid and pyrene was studied and used as a sensor for catechol and its amino-derivatives – dopamine, DOPA and DOPAC. Interestingly D-fructose, D-glucose and phenylalanine did not affect the fluorescence. Theoretical calculations were carried out on the boronate esters and the fluorescence signalling was discussed [29]. A similar set of analytes, namely noradrenaline, dopamine, DOPA and catechol were also investigated using phenylboronic acid and its aminophosphonate analogue **(1)** (Figure 7) [30]. No significant difference in the selectivity across the tested range of the analytes was observed.

#### Fluoride or other anions

The inherent chemical nature of boronic acid as a Lewis acid provides further possibilities to detect strong Lewis bases – such as fluoride anions. A fluoride sensor combining boronic acid and fluorogenic naphthoimidazolium **(2)** was reported [31]. A pyrene based molecular sensor **(3)** was also developed for the detection of fluoride using a biphasic (organic solvent/water) system. Environmentally important levels of fluoride found in drinking water could be determined with this system [32]. An ensemble for the detection of anions was constructed from fluorescent pyrene-based polyelectrolyte with pyridine boronic acid acting as the quencher. The quenched fluorescence of the fluorescent polyelectrolyte was recovered after binding of fluoride or cyanide to the boronic acid [33].

**Figure 8:**
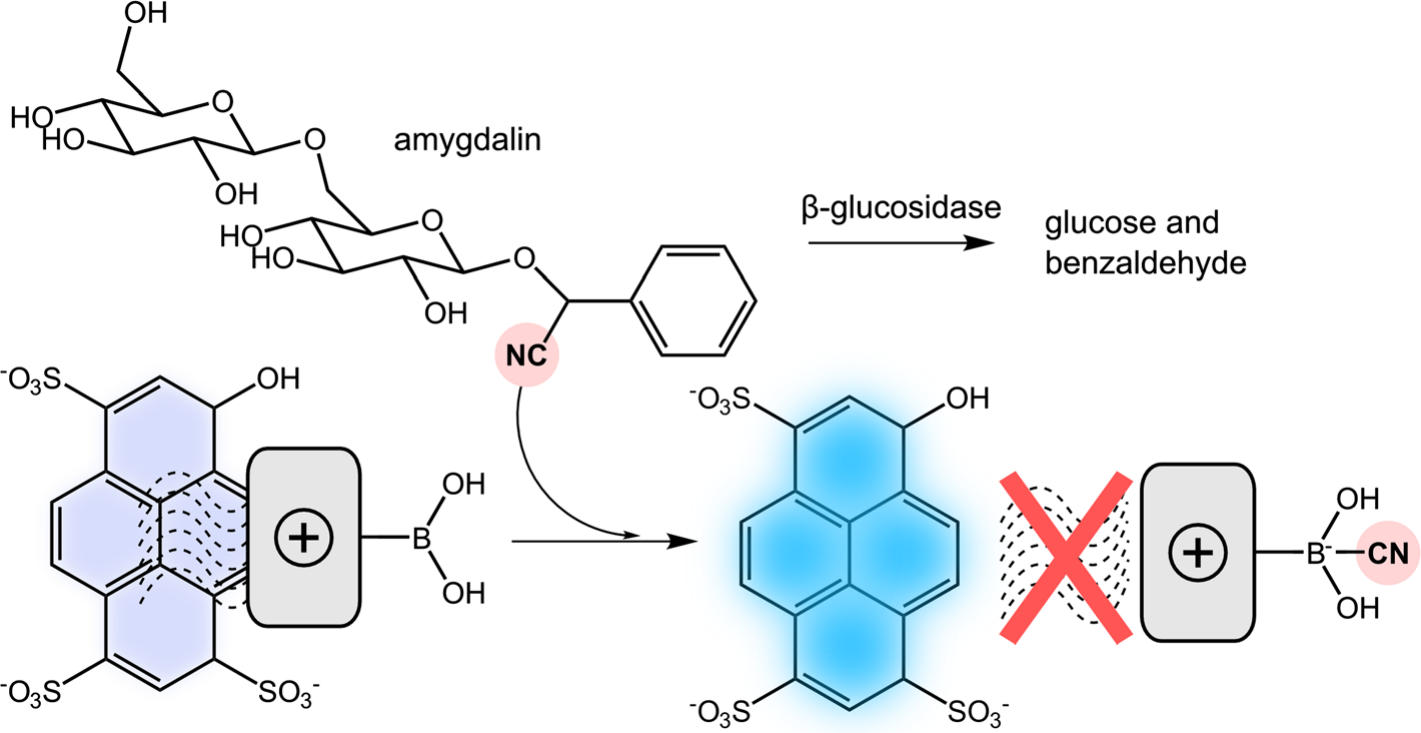
**Fluorescence of a pyrene-based reporter is quenched by uncomplexed boronic acid.** The enzymatically cleaved cyanide group from amygdalin binds to the boronic acid and quenching of the reporter dye is reduced [35].

The cyanide concentration could be quantified using a sensor constructed from phenylboronic acid and methylpyridinium [34]. Amygdalin could be detected by using the enzyme beta-glucosidase and a boronic acid appended viologen – quencher and pyrene-based fluorescent reporter dye (Figure 8). Enzyme cleavage of the amygdalin releases glucose, benzaldehyde and cyanide anion. The generated cyanide anion reduces quenching by the boronic acid quencher and the fluorescence signal is enhanced (recovered) [35].

### Simple surfaces modifications for sensing applications

**Figure 9:**
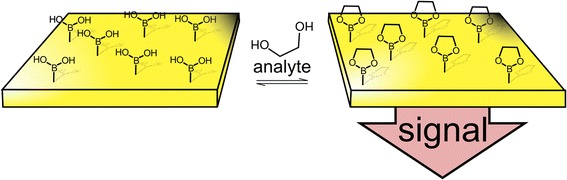
**A heterogeneous detection of diols by means of boronic acid-based sensors assembled in monolayers on a simple surface.**

Advantageous sensing modality is achieved by the attachment of sensor molecules to a surface (Figure 9). The sensing format is then heterogeneous since the binding and detection event take place at the liquid/solid interface. Such systems are desirable since they are suitable for “real” analytical applications.

**Figure 10:**
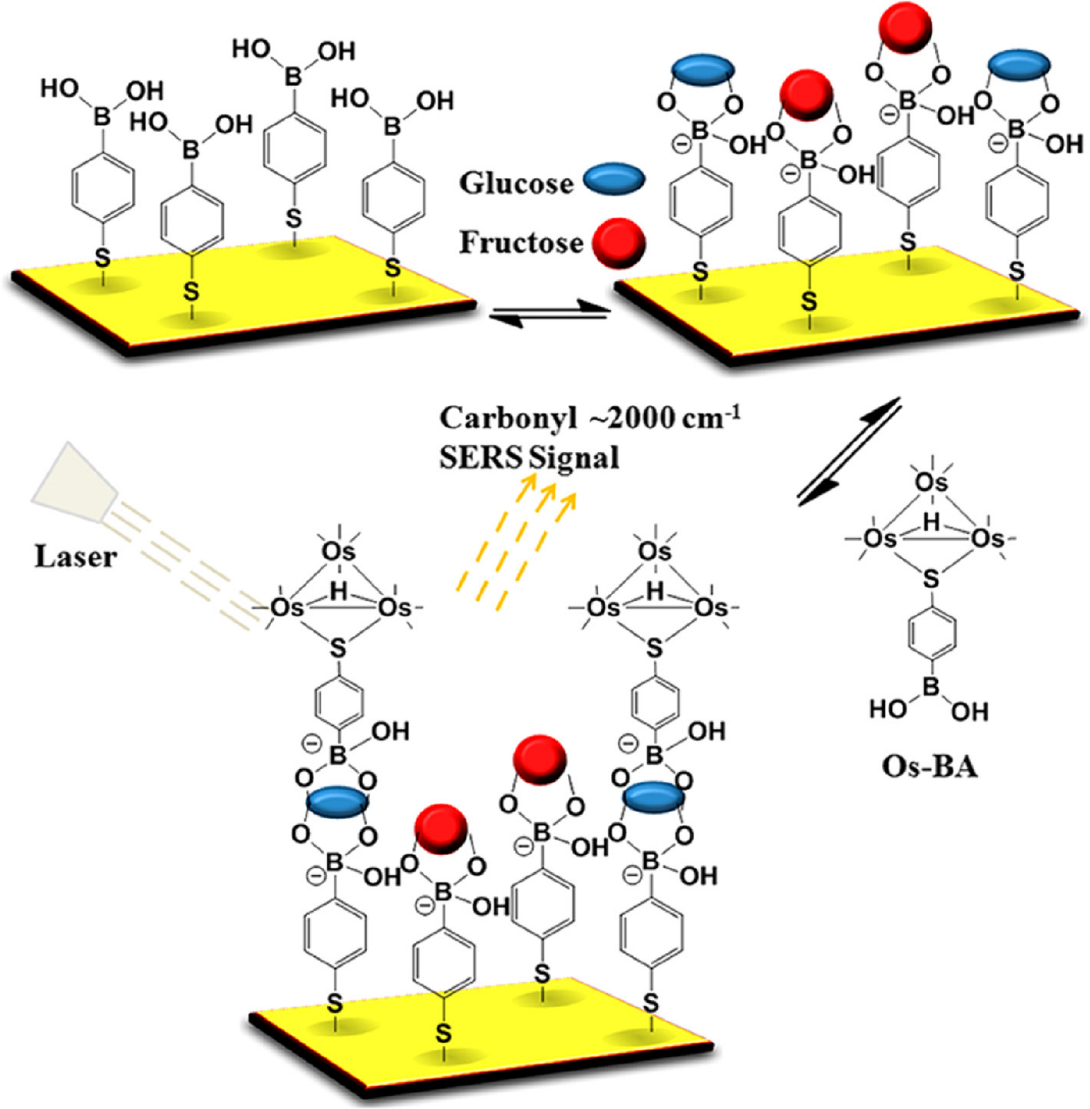
**Surface enhanced Raman scattering sensors for monosaccharides based on boronic acids.** The selectivity for glucose is achieved using 2:1 sandwich-like binding. (Adapted from ref. [40]).

Although fluorescence is more often used for homogeneous systems it can also be used for heterogeneous systems. A fluorescence sensor prepared by the immobilisation of 4-mercaptophenylboronic acid on to a gold CD-trode (compact disc) was used to determine the concentration of monosaccharides down to picomolar levels [36]. However, other signal transduction mechanisms are often used for the read-out from surface sensors. A sensor for hemagglutinin a possible bio-marker for influenza A virus was developed using quartz crystal microbalance (QCM) and surface plasmon resonance (SPR) transducers. The surface of a sensor was modified with aminophenylboronic acid. The boronic acid was then used to anchor sialic acid to the surface, thus, creating a hemagglutinin selective surface [37]. A D-glucose selective SPR sensor **(4)** [38] was developed using a glucose selective bis-boronic acid unit [39] with thiolic functionality, allowing simple attachment to a gold surface. A simple boronic acid was also used for a surface-enhanced Raman scattering (SERS) monosaccharide assay (Figure 10). The boronic acid served both as capturing agent and the triosmium carbonyl boronic acid was employed as a secondary label (in a sandwich assay) [40].

Electrochemical sensing methods are highly advantageous for the transduction of surface confined boronic acid-diol binding events. For instance, the electrochemical impedance of a boronic acid derivatised electrode surface responded to the presence of glucose [41] and other monosaccharides [42] as well as glycated haemoglobin [43].

Modulation of the volt-ampere characteristics of a field effect transistor (FET) due to charge accumulation *i.e*. analyte binding was also utilised. Electrolyte-gated organic FET with input modified by a derivative of phenylboronic acid was used to detect the binding of dopamine [44]. Release of the same analyte was quantified by a silicon nanowire FET after stimulation of PC12 cells by the addition of K^+^ ions [45]. A so-called bio-FET produced using a fluorinated derivative of phenylboronic acid was proposed as a suitable glucose sensor for developing countries since it is simple, cheap and a protein-free analytical device [46].

### Polymeric and other supports for sensing applications

**Figure 11:**
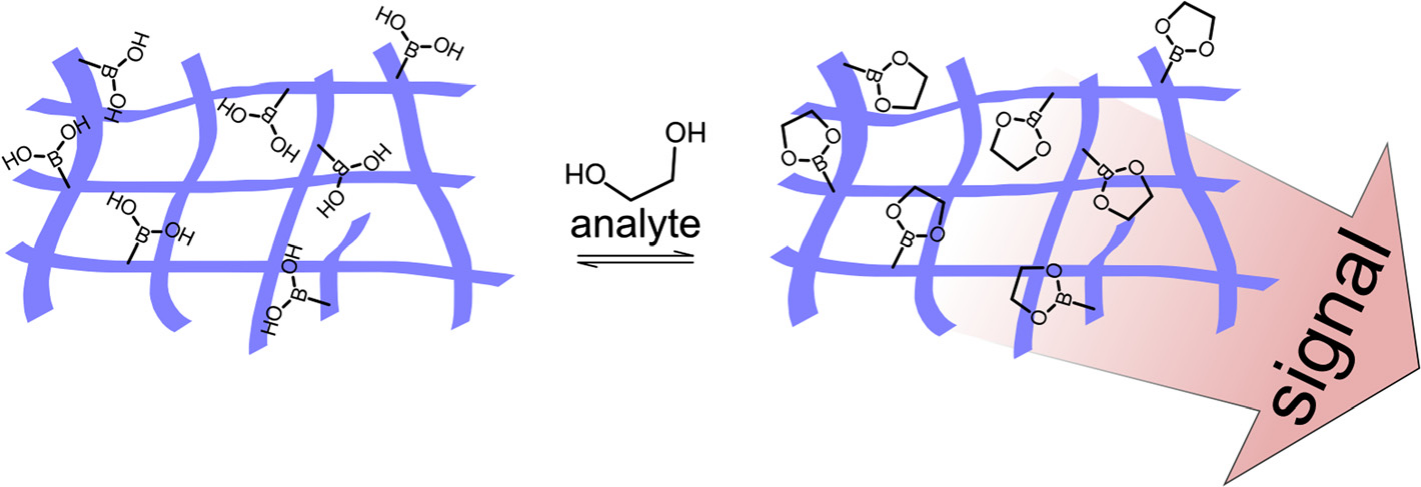
**Detection of diols using boronic acid appended polymer matrix.**

A significant enhancement of binding and hence the performance of a sensor system can be achieved by incorporation of a boronic acid unit into a polymer matrix (Figure 11, Table 1). The inclusion of the molecular sensor into the polymer can help in the development of superior analytical devices, since the polymer imparts many advantages such as improved robustness, sensitivity, handling and biocompatibility. The properties are vital for the development of non-invasive D-glucose sensors [47]. Photonic crystals consisting of a copolymer of acryl amide and styrene gel scaffold with tethered boronic acid was developed to be used for the determination of glucose in tears in the form of contact lenses. Analytical information is obtained from changes of reflected near-infrared light. Photonic crystal can be fabricated via self-assembly. A lamellar block polystyrene scaffold with phenylboronic acid was prepared and its behaviour in the presence of fructose was investigated, a visible colour change from blue to orange was observed [48].

**Table 1:** **Polymeric matrices, transduction method, detection method and analytes for detection systems based on interactions of boronic acid**

***Type of matrix***	***Transduction***	***Detection method***	***Matrix material***	***Analyte***	***Ref.***
Polymer	Optical	Near-infrared	Polyacrylamide	Glucose	[47]
		Fluorescence	Polyacrylamide	Glucose, Oxygen	[49]
		Fluorescence	Polyacrylamide	Glucose	[51]
		Fluorescence	Sepharose	Glucose, Fructose	[54]
		Fluorescence	Polyacrylate	Glucose as effector	[52]
	Electrochemical	Potentiometry, Impedance	PVC membrane	NaF	[58]
		Potentiometry Sequential Injecion Analysis	PVC membrane	Amino acids	[59]
		Potentiometry	electrochemically copolymerised phenylboronic acid/thiophene derivatives	Glucose	[57]
		Impedance	Electropolymerised 3-aminophenylboronic acid	Dopamine	[56]
	Acoustic/mechanic	Viscometry	Polyacrylamide	Glucose	[53]
MIP	Electrochemical	Voltammetry	Polyacrylamide	Dopamine	[60]
		Voltammetry	Poly(aniline-co-anthranilic acid)	Dopamine	[61]
	Optical	Fluorescence	Polymethacrylate/colloidosomes	Isoproterenol	[62]
	Acoustic	Quartz crystal microbalance	Polyacrylate	Mannose, Ig M	[63]
Photonic crystal	Optical	Diffraction	Poly(acrylamide-co-methacrylate)	Glucose	[64]
		Diffraction	Polystyrene	Fructose	[48]
Vesicles	Optical	Fluorescence	N-Alkyl-3-boronopyridinium surfactants	Monosaccharides	[55]

Sensing of oxygen and glucose was performed under biological conditions. A sensor consisting of a polyacrylamide-based matrix with three different fluorescent probes for oxygen, glucose and one reference channel was used to detect the consumption of glucose and oxygen by bacterial and mammalian cells [49]. A D-glucose sensitive probe was based on the Shinkai bis-boronic acid derivative [50] and for oxygen sensing a modified perfluoro-platinum porphyrin was used.

**Figure 12:**
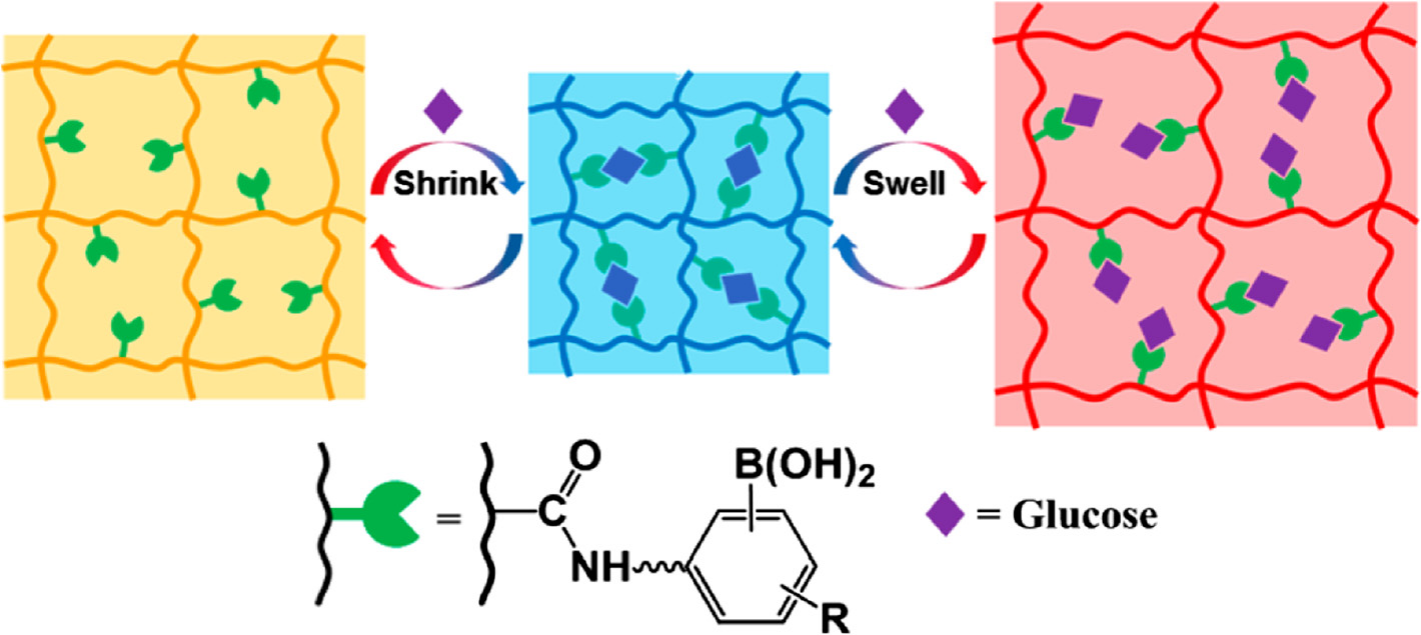
**Concentration dependent shrinking or swelling of the polymer.** (Adapted from ref. [52]).

Changes of the gel diameter (swelling, shrinking) can be used for detection and also for therapeutic purposes. An acrylamide gel containing boronic acid and low-cost fluorescent dye, Bordeaux R was used for a detection of glucose. The Bordeaux R interacts with amide groups of the gel and causes the gel to shrink. On exposure of the gel to D-glucose the polymer network swells and distorts the π-π stacking in the assemblies of dye molecules, thus modulating the fluorescence intensity of the dye molecules embedded in the gel [51]. The therapeutic application can be exemplified *via* a polyacrylic-boronic acid gel which shrinks or swells depending on the D-glucose concentration (Figure 12). Such systems could be used for the D-glucose controlled release of insulin [52].

The viscosity of a polyacrylamide scaffold was used for analytical purposes. Changes in viscosity due to fluctuations in D-glucose concentrations were detected using microelectromechanical system (MEMS) [53].

A sepharose gel with appended boronic acid functionality (click chemistry) exhibited dose-dependent fluorescence intensity upon binding with D-glucose and D-fructose [54]. The fluorophore ARS was used to characterise the binding properties of amphiphilic boronic acid vesicles in a detection system for diols [55].

Electropolymerisation is a technique for the tightly controlled and defined preparation of polymeric structures. Electropolymerised 3-aminophenylboronic acid was used for the impedimetric detection of dopamine [56] and a co-polymer of phenylboronic acid with thiophene was used for the potentiometric detection of D-glucose [57].

Furthermore, a boronic acid derivative was incorporated into a PVC membrane and integrated into an electrode that was employed for potentiometric detection of sodium and impedimetric detection of fluoride ions. Fluoride ions interacting with the boron atom served as an effector [58]. The so-called electronic tongue combining a potentiometric sensor array with sequential injection analysis (SIA) was employed for the determination of four different amino acids - phenylalanine, tyrosine, ornithine and glutamic acid. The individual sensors were based on polymeric membranes (plasticised PVC) containing 4-octyloxyphenylboronic acid as an ionophore [59].

#### Molecular imprinted polymers (MIP)

Many different materials and methods have been used for the preparation of diol sensors based on molecularly imprinted polymers. The boronic acid unit has specific receptor abilities suitable for MIP technology.

A MIP electropolymerised onto the surface of an electrode was used for the quantification of dopamine [60], imprinted poly(aniline-co-anthranilic acid) as the supporting material together with phenylboronic acid was able to detect dopamine in human plasma [61]. Molecularly imprinted nanoparticles – colloidosomes provided with boronic acid were used for quantification of the β-adrenergic receptor agonist, isoproterenol. Colloisodomes were synthesised by means of click chemistry [62]. Acrylate-based MIP immobilised on a QCM sensor was used for the selective recognition of immunoglobulin M (IgM) and mannose over fructose [63]. MIP technology was also used for preparation of a photonic crystal technology with sensitivity towards glucose [64].

### Electrophoresis

**Figure 13:**
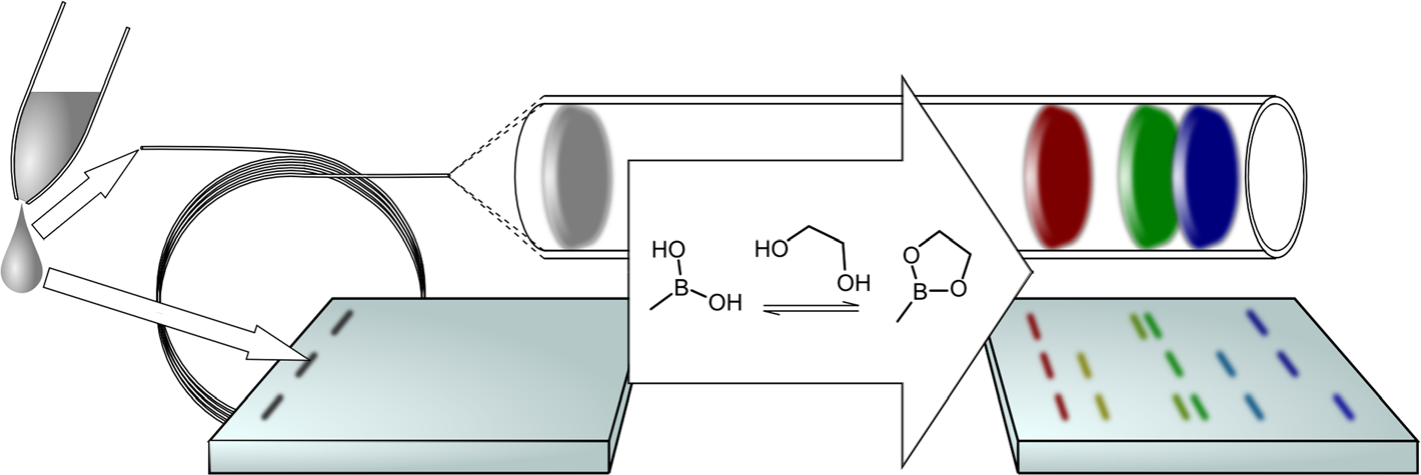
**Utilisation of specific boronic acid-diol interactions for a capillary or gel electrophoresis.**

Boronic acid is well suited for affinity based detection and separation techniques such as electrophoresis (Figure 13). Capillary electrophoresis was established and validated to probe the interactions between boronic acids and *cis*-diol-containing biomolecules. Interaction of 14 different boronic acids and 5 typical monosaccharides was comprehensively studied and kinetic data was obtained, however, deeper discussion concerning the nature of the interactions is lacking [65]. Furthermore electrophoresis combined with boronic acid derivatives seems promising for the analysis of monosaccharides [66], glycoproteins - detection of glycated haemoglobin [67] and as a powerful proteomic tool with the ability to identify potential biomarkers for age-related diseases [68].

### Micro and nanoparticles

**Figure 14:**
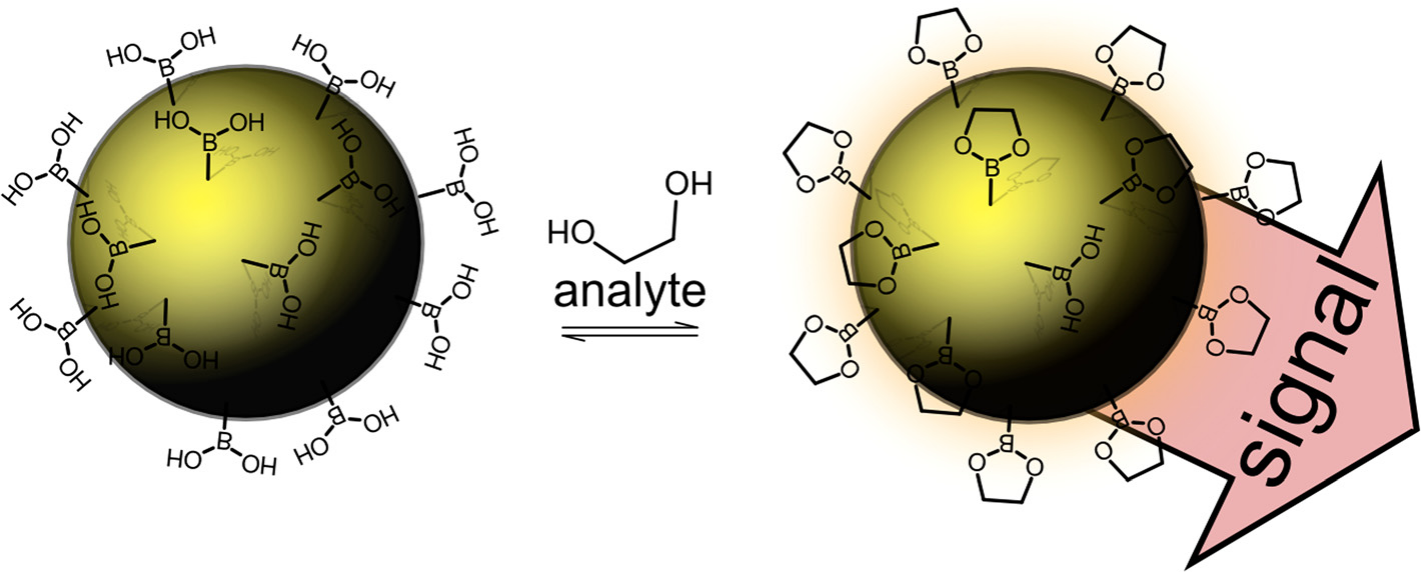
**Selective detection of diols using nanoparticles modified with boronic acids.**

The unique abilities of micro- and especially nanoparticles can be enhanced by incorporation of boronic acids to the outer surface (Figure 14). For example, the abilities to prepare diverse sensor materials by the modification of carbon nanotubes were reviewed [11]. An approach combining boronic acid and single-walled carbon nanotubes (SWNT) resulted in near-infrared (NIR) optical glucose sensors, that could be used for long-term *in vivo* continuous glucose monitoring. The modulation of SWNT fluorescence upon glucose binding and potential *in vivo* applications for clinical use was discussed [11].

Carbon is a popular building material used in many “nanoapplicaitons”. A transistor made of carbon nanotubes functionalised with pyrene-1-boronic acid was sensitive to concentrations of D-glucose in dilute solution at the nM level [69]. D-Glucose, D-fructose and D-mannose were determined in fruit juices using glassy carbon electrodes modified with graphene oxide and 4-aminophenylboronic acid [70]. The interaction of boronic acid modified water-insoluble carbon nanoparticles with the aromatic diol caffeic acid was measured using a pyrolytic graphite electrode, the interactions could be described using Langmuirian binding kinetics [71]. Quantum dots (QD) are another promising material for analytical purposes, especially for clinical applications [72]. 3-aminophenylboronic acid functionalised graphene quantum dots together with microdialysis were used for the selective detection of D-glucose in rat striatum. The fluorescence of the QD was observed upon the addition of saccharides.

Gold nanoparticles are the subject of several interesting works from 2013. The combination of mercaptophenylboronic acid, gold nanoparticles, graphene and enzyme glucose oxidase resulted in a material able to detect glucose [73]. The same analyte was detected using a system consisting of gold nanoparticles functionalised with calix [4] arene/phenylboronic acid [74]. Gold nanoparticles modified with 4-mercaptophenylboronic acid were used to detect tyrosinase activity. The system was used to quantify surface bound catechol which was produced by enzymatic oxidation of phenol initially present in the system [75]. In another study investigating potential cancer marker, microRNA was captured by a DNA probe on the electrode surface. 4-mercaptophenylboronic acid appended gold nanoparticles and catechol functionalised gold nanoparticles (as an electrochemical label) were used in this system [76].

**Figure 15:**
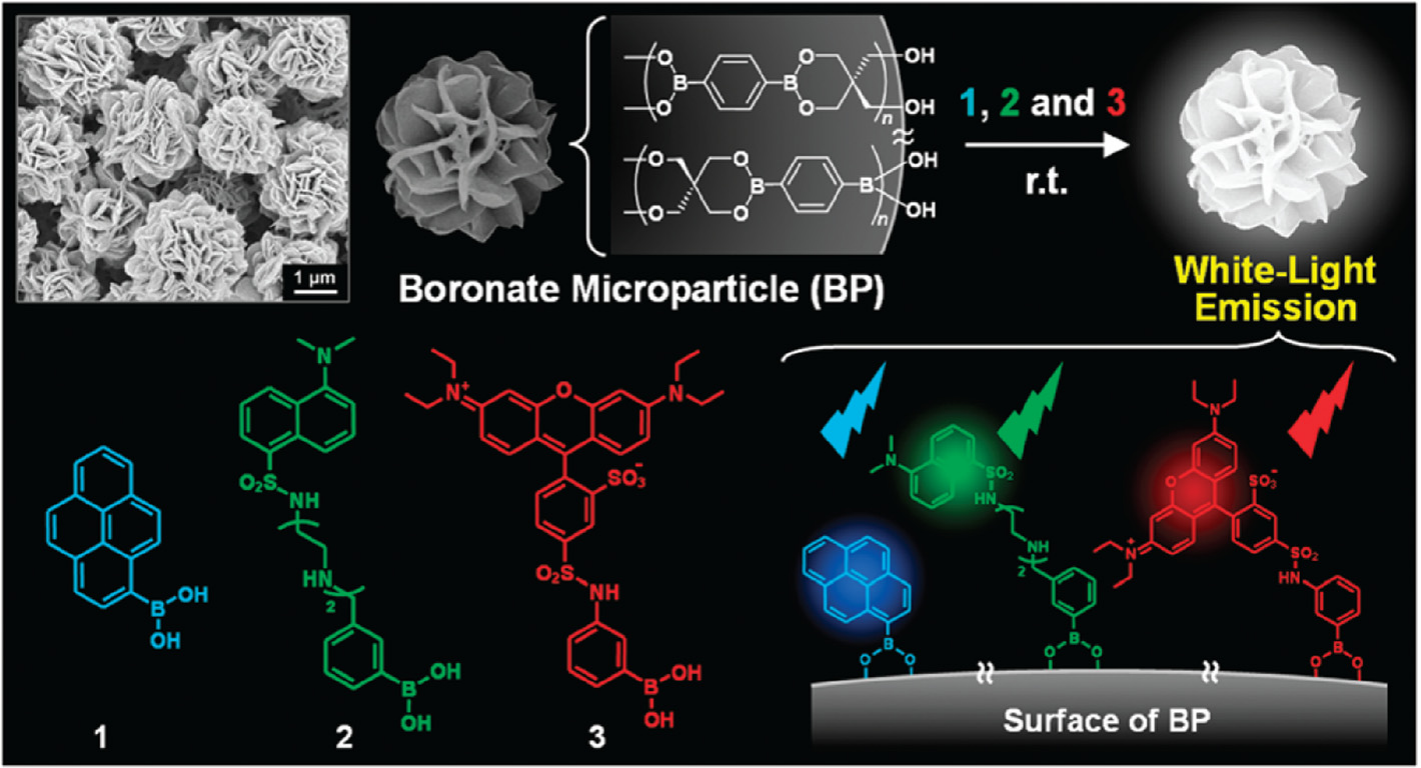
**Preparation of water-dispersible white-light emitting boronate microparticles together with the structure of reporter dyes employed.** (Adapted from ref. [77]).

A sophisticated and elegant system using three different fluorophores to produce white light was developed (Figure 15). Three flourophores were immobilised by means of boronic acid/diol interactions. After interaction with different concentrations of analyte, Cu^2+^ ions, the emission was quenched thus altering the colour [77].

### Detection of reactive oxygen species (ROS)

**Figure 16:**
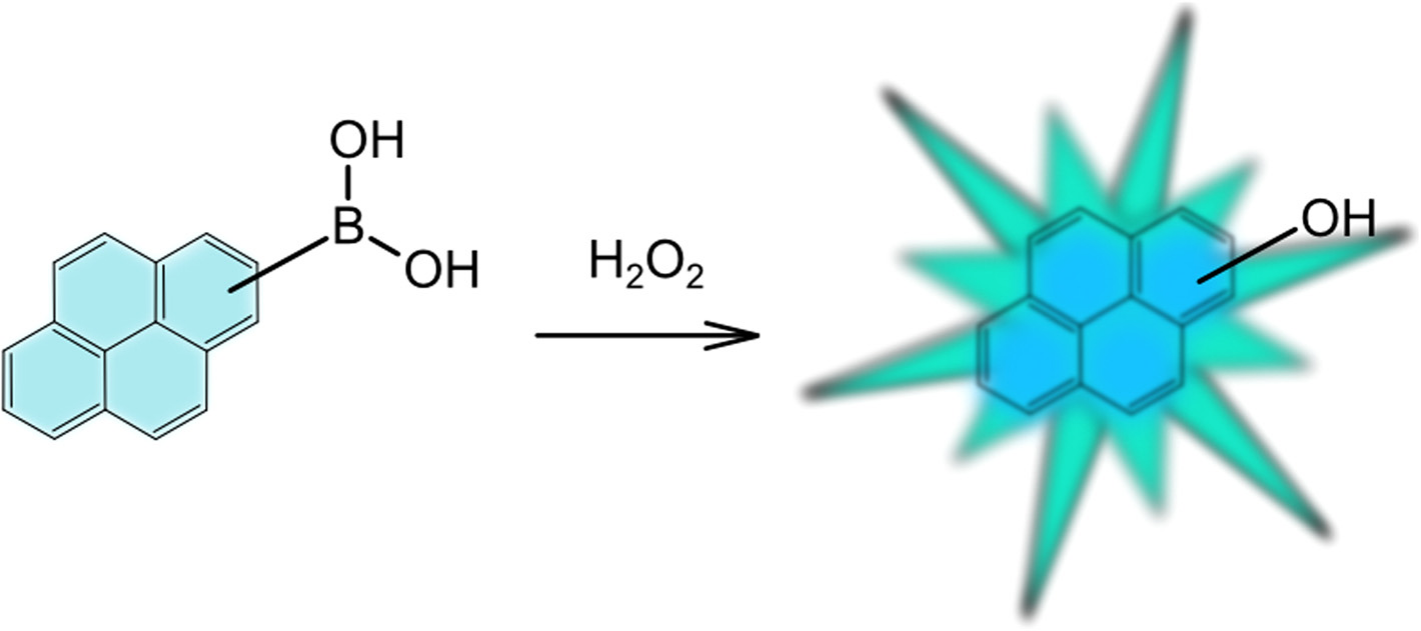
**Boronic acid-based molecular sensors for the homogeneous optical detection of reactive oxygen species.**

**Figure 17:**
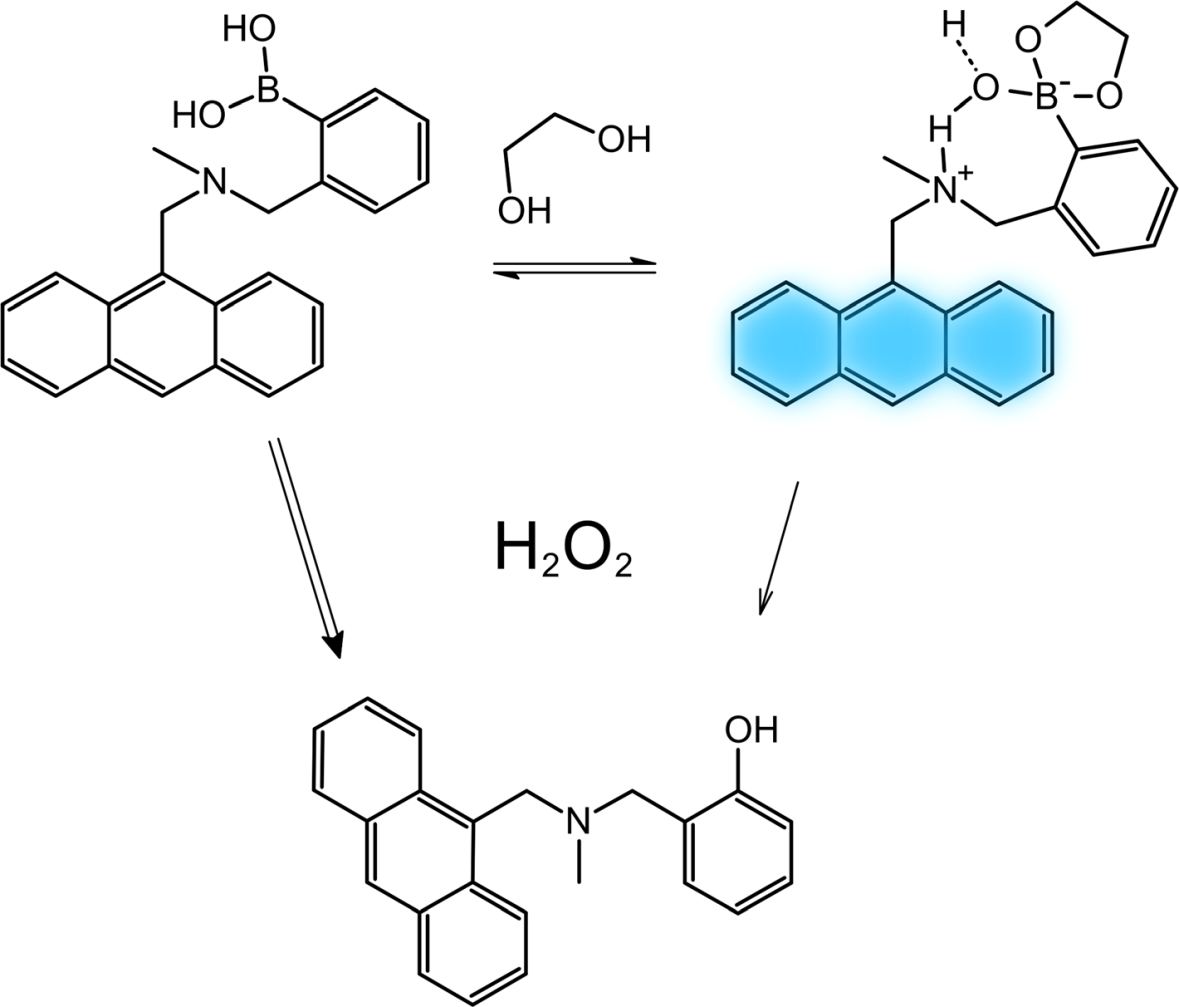
**The cleavage of the boronate group from fluorogenic derivative used for the detection of reactive oxygen species (H**_**2**_ **O**_**2**_ **)** [78]**.**

A relatively recent sensing area using boronic acids are chemosensors for the detection of reactive oxygen species (ROS) (Figure 16). ROS play an important role in cellular signalling, immune response and take part in many pathological processes. Oxidative cellular damage is also, related with illnesses such as cancer and Alzheimer’s disease. A recently reported sensor molecule detects ROS *via* the cleavage of a boronate group from an ester of a fluorogenic boronic acid (Figure 17) [78].

Peroxynitrate and hypochlorite anions are also important members of the ROS family. Peroxynitrate was detected using a sensor combining boronic acid and luminescent luciferin [79]. Peroxynitrate was also, detected using the genetically encoded fluorescent protein with appended boronic acid [80]. While, a water soluble styrylquinolinium boronic acid **(5)** was used for the selective sensing of hypochlorite anion [81].

### Electrochemical detection

**Figure 18:**
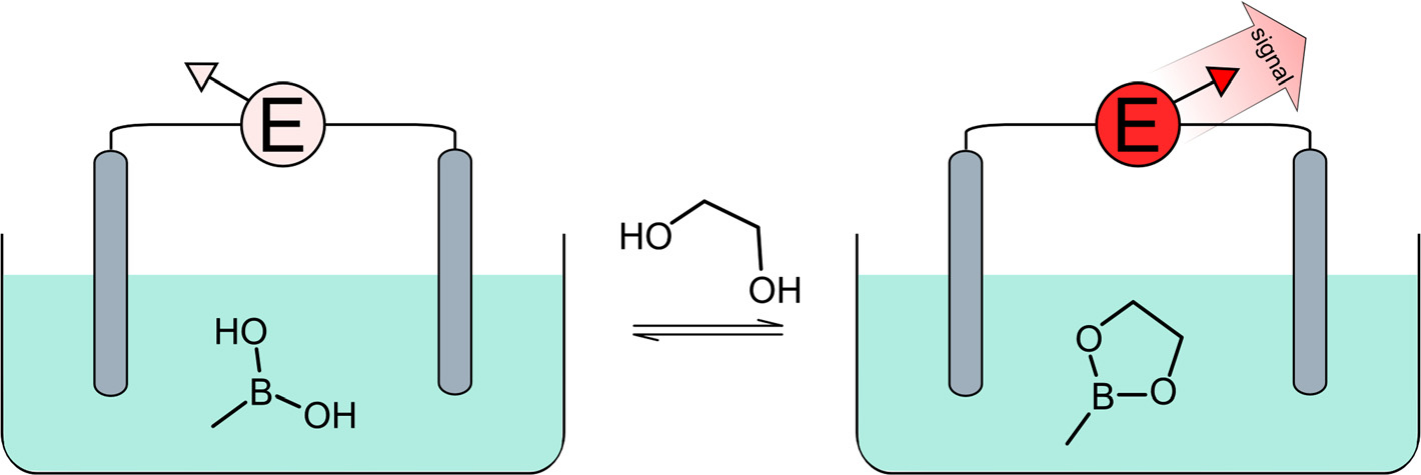
**Electrochemical detection based on the boronic acid-diol interaction.**

Electrochemical methods (Figure 18) are fewer in comparison to optical methods, however, they are highly desirable for all surface confined sensing applications. For example, heterogeneous applications which take place at the interphase of solution and electrode modified with boronic acids. In fact the majority of the previously discussed surface related detection systems use electrochemical methods for the transduction of the out-put signal. Electrochemical impedance spectroscopy was used for the sensing at a surface [41–43] and also in polymers [56,58]. Changes in another electrochemical property, the surface potential can also be followed using classical potentiometry [57,58] and using this technique makes the construction of an electronic tongue feasible [59].

As previously discussed many heterogeneous sensing applications utilise field effect transistors with the modulation of their electronic characteristics [44–46,69]. Importantly, the heterogeneous process of interaction of surface-bound boronic acid with diol can be improved by inclusion of nanoparticles [69–71,75,76].

Although electrochemical methods are from their very nature heterogeneous, homogeneous processes can also be probed using these techniques. This type of electrochemical detection can be based on either electrochemically active derivatives of boronic acid evolved from e.g. ferrocene [82] and other typical electrochemical labels or the electroactivity of the diol molecule. Voltammetric techniques, e.g. cyclic and differential pulse voltammetry (CV and DPV) with their versatility are the method of choice. DPV performed on glassy carbon electrodes with graphene-4-aminophenyl boronic acid have been used for the detection of D-fructose, D-glucose and D-mannose [70]. Both CV and DPV were used for the detection of molecules with biological relevance – dopamine [60,61], caffeic acid [71], tyrosinase [75] and microRNA [76,83]. Also, the Wang ARS displacement assay [17] was modified and used for the electrochemical detection of saccharides [84]. In this case dopamine was employed as the reporter molecule which is being displaced in the course of measurement.

### Other systems and applications

**Figure 19:**
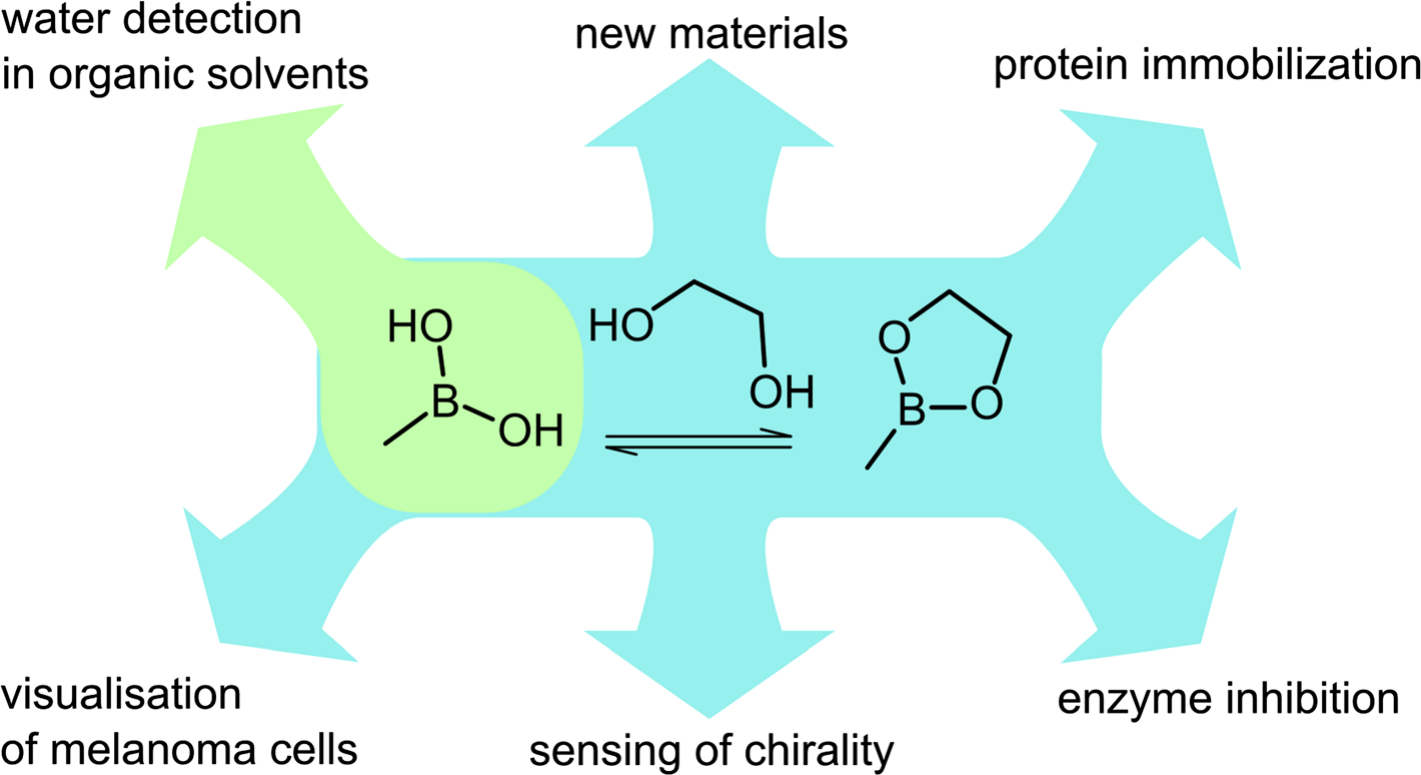
**The many diverse and potential applications for boronic acid groups.**

**Figure 20:**
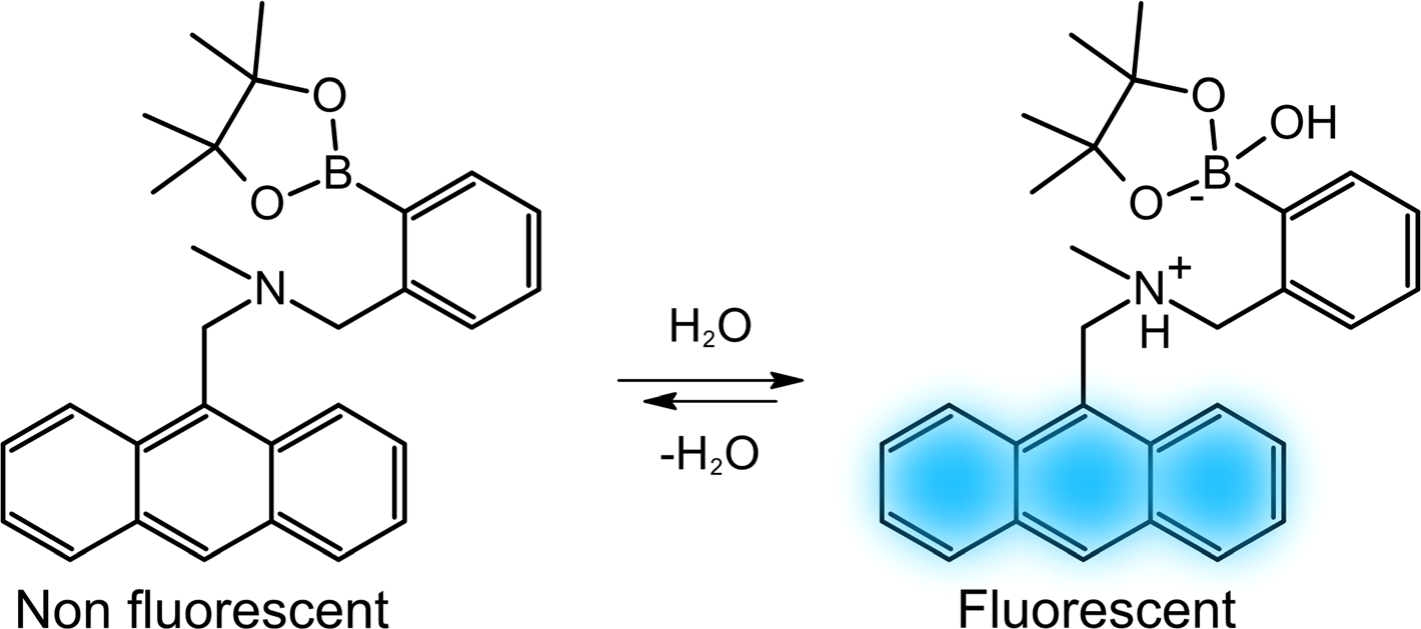
**Fluorescence detection of water in organic solvents [** 88 **]** **.**

Boronic acids play a crucial role not only in organic synthesis, but they are vital in many sensor applications and also find use in a range of therapeutic applications (Figure 19). Therefore, it is not surprising then that the well described behaviour of boronic acid is still at the fore-front of research by the scientific community. In order to understand this vital interaction better, the behaviour of arylboronic acids was systematically investigated using ESI-MS [85]. A detailed computational investigation of the ability of boronic acids to bind saccharides was carried out [86]. The important role of the interaction between an *ortho*-aminomethyl functionality and boron was studied [87]. Furthermore this functionality was utilised for quantification of trace amount of water in organic solvents (Figure 20). Fluorescence from the anthracene-boronic acid esters was used to measure the amount of water present [88].

Self-assembled microspheres were prepared from benzene-1,4-diboronic acid and pentaerythritol [77]. Since polyols and especially saccharides are abundant structures in biological systems, they can be employed with boronic acids in many biocompatible applications. Chaperonin-based boronic acid embedded nanocarriers displayed selective guest release on changes in the concentration of ATP [89]. Boronic acid modified nanocarriers lead to the enhanced uptake by human epithelial carcinoma cells. Therefore, the construction of biomolecular machinery with the aim of localised delivery and release of therapeutic agents was suggested.

**Figure 21:**
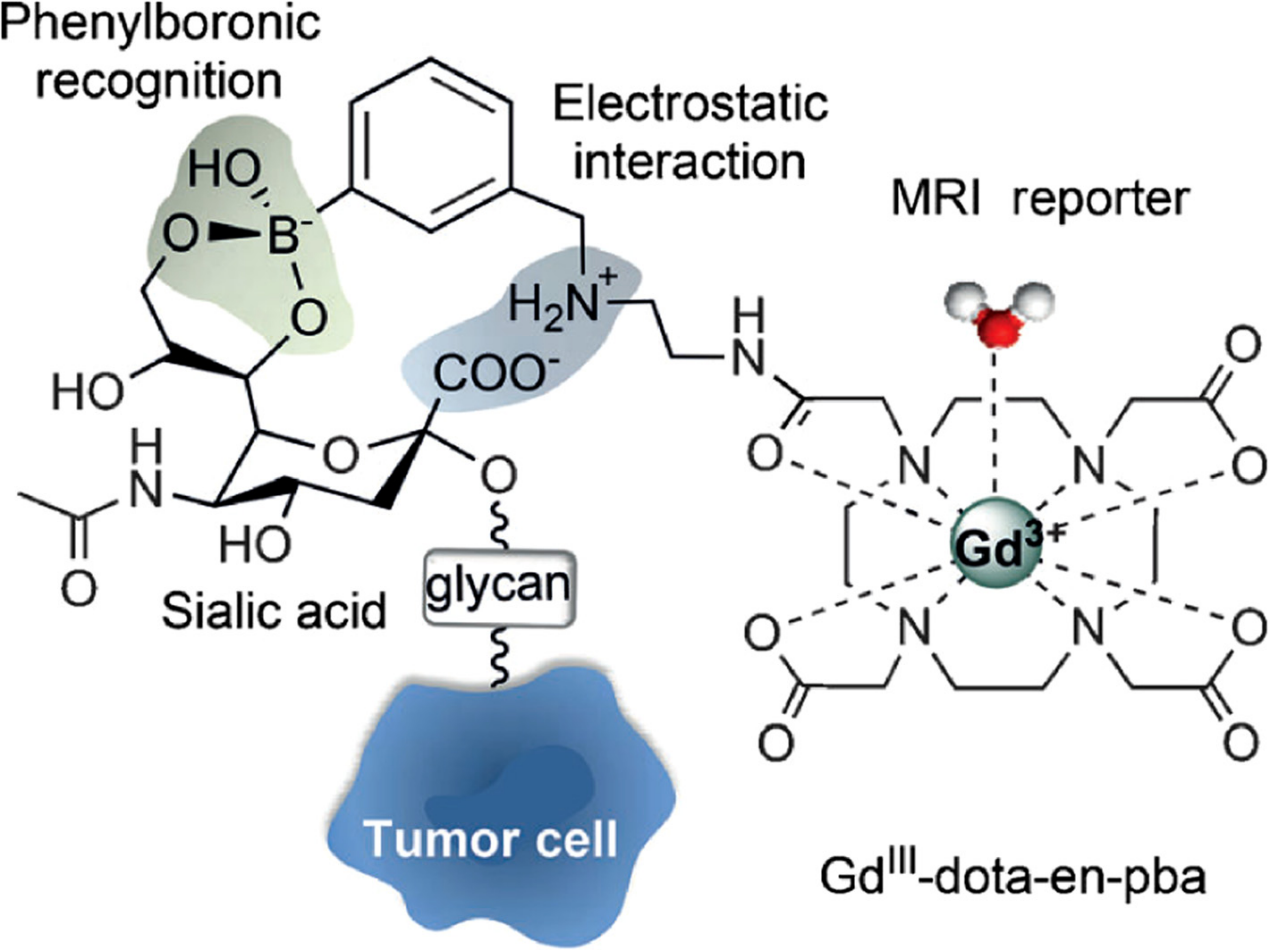
**Magnetic resonance imaging (MRI) of tumour cells.** The over-expressed sialic acid on the cell membrane is recognised by the boronic acid-based MRI agent. (Adapted from ref. [95]).

Boronic acids were used for the direct immobilisation of proteins by the interaction with the glycosylated part of the protein structure [90–92] or by means of immobilisation of their particular coenzymes [93,94] for their detection or for the detection of other analytes. Boronic acid derivatives were employed for the visualisation of tumour cells (Figure 21). Interaction of boronic acid-magnetic resonance imaging (MRI) probe (Gadolinium based chelate) with overexpressed sialic acid on the surface of cells was used to locate melanoma cells [95].

Boronic acids have also been used to investigate the interactions and functions of proteins. The rationally designed boronic acid derivative (1-tridecylboronic acid) inhibited siderophore biosynthetic enzymes, and it was proposed as a possible treatment to control P. aeruginosa infections. It is assumed that the derivative mimics an important reaction transition state [96].

A sophisticated ensemble of 2-formylphenylboronic acid and enantiopure BINOL for the detection of enantiomeric purity of chiral hydroxylamines was reported. The resulting mixture of diastereomeric nitrono-boronate esters reflects the enantiopurity of the parent hydroxylamine [97]. Finally, a sensor for cationic surfactants based on polydiacetylene oxyphenylboronic acids was developed and tested [98].

## Conclusions

The utilisation of boronic acid in many diverse areas of chemistry and biology were reviewed. One of the most common applications of boronic acids (BAs) is the detection of diols and anions. Some interesting examples of specific molecular receptors, using novel sensing methods were reported (optical or electrochemical). In particular, sensing applications were developed from the direct immobilisation of the boronic acid based receptors to a surface or as part of a polymeric matrix. Moreover, suitably modified polymeric matrices were used for functional materials, for example the controlled release of insulin. The properties of BAs can be enhanced or modified by anchoring them to a surface of micro or nanoparticles. There is a growing interest the use of BAs in the sensing and labelling of biologically important molecules such as proteins, and the detection of reactive oxygen species (ROS).

From this review it is hoped that the reader will discover that “Boronic acids” have many uses including synthetic chemistry (not included in this review), biological labelling, therapeutic applications (medicinal treatment), protein manipulation (immobilisation), affinity separation and much more. It is important to note that the area of BA research is still open for the development of new ideas, including the development of new detection methods and novel functional materials, towards the development of systems with impact for real world applications.

## Abbreviations

ARS: Alizarin Red S
BINOL: 1,1'-bi-2-naphthol
BODIPY: 4,4-difluoro-4-bora-3a,4a-diaza-s-indacene
CV: Cyclic voltammetry
DMSO: Dimethyl sulfoxide
DOPA: 3,4-dihydroxyphenylalanine
DOPAC: 3,4-dihydroxyphenylacetic acid
DPV: Differential voltammetry
ESI-MS: Electrospray ionisation – mass spectroscopy
FET: Field effect transistor
FRET: Förster resonance energy transfer
MEMS: Microelectromechanical system
MIP: Molecular imprinted polymers
MRI: Magnetic resonance imaging
NIR: Near-infrared
NMR: Nuclear magnetic resonance
PVC: Polyvinylchloride
ROS: Reactive oxygen species
SERS: Surface enhanced Raman spectroscopy
SPR: Surface plasmon resonance
SWNT: Single-walled carbon nanotubes
QCM: Quartz crystal microbalance
QD: Quantum dots
